# Impact of protocatechuic acid on alleviation of pulmonary damage induced by cyclophosphamide targeting peroxisome proliferator activator receptor, silent information regulator type-1, and fork head box protein in rats

**DOI:** 10.1007/s10787-023-01156-6

**Published:** 2023-03-06

**Authors:** Abeer Salama, Rania Elgohary, Mohamed M. Amin, Sahar Abd Elwahab

**Affiliations:** 1 Pharmacology Department, Medical Research and Clinical Studies Institute, National Research Centre, 33 El Buhouth St. (Former El-Tahrir St.), Dokki, Cairo, 12622 Egypt; 2 Narcotics, Ergogenics and Poisons Department, Medical Research and Clinical Studies Institute, National Research Centre, 33 El Buhouth St. (Former El-Tahrir St.), Dokki, Cairo, 12622 Egypt; 3 Medical Biochemistry and Molecular Biology Department, Faculty of Medicine Cairo University Al Kasr Al Aini, Old Cairo, Cairo Governorate, Egypt

**Keywords:** Protocatechuic acid, Pulmonary damage, Inflammation, PPARγ, Cyclophosphamide, FoxO-1

## Abstract

Cyclophosphamide (CP) is a chemotherapeutic agent that causes pulmonary damage by generating free radicals and pro-inflammatory cytokines. Pulmonary damage has a high mortality rate due to the severe inflammation and edema occurred in lung. PPARγ/Sirt 1 signaling has been shown to be cytoprotective effect against cellular inflammatory stress and oxidative injury. Protocatechuic acid (PCA) is a potent Sirt1 activator and exhibits antioxidant as well as anti-inflammatory properties. The current study aims to investigate the therapeutic impacts of PCA against CP-induced pulmonary damage in rats. Rats were assigned randomly into 4 experimental groups. The control group was injected with a single i.p injection of saline. CP group was injected with a single i.p injection of CP (200 mg/kg). PCA groups were administered orally with PCA (50 and 100 mg/kg; p.o.) once daily for 10 consecutive days after CP injection. PCA treatment resulted in a significant decrease in the protein levels of MDA, a marker of lipid peroxidation, NO and MPO along with a significant increase in GSH and catalase protein levels. Moreover, PCA downregulated anti-inflammatory markers as IL-17, NF-κB, IKBKB, COX-2, TNF-α, and PKC and upregulated cytoprotective defenses as PPARγ, and SIRT1. In addition, PCA administration ameliorated FoxO-1 elevation, increased Nrf2 gene expression, and reduced air alveoli emphysema, bronchiolar epithelium hyperplasia and inflammatory cell infiltration induced by CP. PCA might represent a promising adjuvant to prevent pulmonary damage in patients receiving CP due to its antioxidant and anti-inflammatory effects with cytoprotective defenses.

## Introduction

Pulmonary damage can result from sepsis, trauma, microbial infection, ischemia/reperfusion, or some drugs that induce acute respiratory failure with a mortality rate of approximately 40% (Righetti et al. 2018). The pathophysiology of pulmonary damage is characterized by infiltration of polymorphonuclear cells (PMNs) and epithelial integrity disruption which leads to interstitial edema and alveolar collapse (Castillo et al. 2015). Macrophages and neutrophils are vital mediators in stimulating the inflammatory reaction, controlling oxidative stress, and fibroblast function in pulmonary damage. In addition, macrophages and neutrophils release pro-inflammatory cytokines, such as tumor necrosis factor (TNF)-α, interleukin-1beta (IL-1β), and IL-6 (IL-6), as well as transforming growth factor beta (TGF-β) (Blondonnet et al. 2016). These inflammatory cytokines suppressed by the peroxisome proliferator-activated receptors (PPARs) hormone in the lung (Kaplan et al. 2005). In addition, Sirtuin has a critical role in regulating macrophage mitochondrial bioenergetics (Kurundkar et al. 2019). Silent information regulator type-1 (SIRT1) controls gene expression for apoptosis, stress responses, and cellular senescence. SIRT1 exhibits antioxidative, antiangiogenic, antitumorigenic, and anti-inflammatory effects (Li et al. 2013). It has received much attention due to its role in resistance to oxidative stress, which is one of the mechanisms involved in the SIRT1/Forkhead box Protein (FOXOs) pathway (Mahlooji et al. 2022). FoxO1 is the main transcription factor of fundamental cellular processes, mainly regulating oxidative stress, cell proliferation, inflammation, immune homeostasis, and cell apoptosis (Arcidiacono et al. 2018).

Cyclophosphamide (CP), a synthetic alkylating agent, treats autoimmune diseases and cancer, as well as prevents organ transplantation rejection (Emadi et al. 2009). Despite its wide range of clinical applications, it produces multiorgan damage, which in turn causes severe morbidity and mortality (Haubitz 2007). Most reports focused on its hepatotoxicity (SNYDER et al. 1993), cardio- and gonadotoxicity (Swamy et al. 2013), and lung toxicity (Alsemeh and Abdullah 2022). It has been proposed that CP administration causes cellular respiration impairment through damage to mitochondria (Souid et al. 2003), which leads to liberation of reactive-oxygen species (ROS) (Suddek et al. 2013; Zarei and Shivanandappa 2013) and triggers the nuclear factor-κB (NF-κB) inflammatory pathway (Hamsa and Kuttan 2010).

Protocatechuic acid (PCA), is a polyphenolic compound, present in many vegetables and nuts (Vitaglione et al. 2007). It has anti-inflammatory, antioxidant, and hepatoprotective properties, mainly by inhibiting stress signal transduction as it suppresses Cyclooxygenase-2 (COX-2), nitric oxide synthase, and myeloperoxidase (MPO) in CCl4-induced liver injury (Hsu et al. 2009; Liu et al. 2002). In addition, it has a protective effect in lung injury (Alsharif et al. 2021).

In this regard, the current study investigates the effect of protocatechuic acid on PPARs/SIRT/FOX to mitigate CP-induced pulmonary damage in rats.

## Materials and methods

### Animals

Adult male Wister albino rats (150–200 g) were provided by the Animal House of the National Research Centre (Cairo, Egypt). The rats were group-housed with free access to standard laboratory rodent chow and water under temperature−light-controlled conditions (24 ± 2 °C under a 12 h light/dark cycle). The animal experiments were performed in according to recommendations in the Guide for the Care and Use of Laboratory Animals of the National Institutes of Health (NIH No. 85:23 revised 1985) in accordance with the guidelines of the Institutional Animal Ethics Committee (Medical Research Ethics Committee (MREC) of the NRC, Cairo, Egypt.

### Drugs, chemicals, and kits

Cyclophosphamide and protocatechuic acid were purchased from (Santa Cruz, CA, USA). Glutathione (GSH), malondialdehyde (MDA), nitric oxide (NO), and catalase colorimetric kits were determined using Biodiagnostic kit, Cairo, Egypt. Sirtuin 1 (SIRT1) was determined using ELISA kits procured from (SunRed Biotech Co., Ltd, China). Tumor necrosis factor alpha (TNF-α), Interleukin-17 (IL-17), nuclear factor kappa B (NF-κB), inhibitor of nuclear factor kappa beta kinase subunit beta (IKBKB), proxisome proliferator-activated receptor gamma (PPARγ), cyclooxegnase-2 (COX-2), protein kinase C (PKC), and myeloperoxidase (MPO) were determined using ELISA kits procured from Sunlong Biotech Co., Ltd, China.

### Experimental design

Wister albino male rats were randomly allocated into four groups (*n* = 8) as follows: Control group: Rats were injected with a single i.p injection of normal saline and received normal saline orally for 10 consecutive days. CP group: Rats were injected with a single i.p injection (200 mg/kg) (Saghir et al. 2020). Protocatechuic acid groups: Rats were administered protocatechuic acid (50 &100 mg/kg orally) (Krzysztoforska et al. 2019) once daily for 10 consecutive days after CP injection.

### Biochemical analysis

At the end of the experimental period, the animals were sacrificed by decapitation; the lung from each rat was immediately dissected out, washed with ice‐cooled physiological saline, and homogenized in phosphate-buffered saline (PBS)(pH 7.4) as 20% (w/v) for the biochemical measurements of GSH, MDA, NO, and catalase were determined using Biodiagnostic colorimetric kits. Moreover, SIRT1, IL-17, NF-κB, IKBKB, PPARγ, COX-2, TNF-α, MPO, and PKC were determined using ELISA kits. The other lung was kept for histopathological assessment.

#### Estimation of GSH, MDA, NO, and catalase

GSH lung content was estimated according to the method that depends on the fact that protein and nonprotein SH-groups (mainly GSH) react with Ellman’s reagent [5,5′-dithio-bis(2 nitrobenzoic acid)] to form a stable yellow color of 5-mercapto-2-nitrobenzoic acid, which can be measured colorimetrically at 412 nm. In order to determine the GSH level in tissue, precipitation of protein SH-groups before the addition of Ellman’s reagent is necessary using precipitating solution (5% sulfosalicylic acid in bi-distilled water) (Basha et al. 2020).

Lung homogenate was used for the assessment of lipid peroxidation (LPO) as MDA and catalase activity according to the methods described by Ohkawa et al and Salama and Elgohary (2021), (Ohkawa et al. 1979; Salama and Elgohary 2021), respectively. In addition, NO was measured according to the method described by Tarpey et al. 2004. Briefly, nitrate was reduced to nitrite using the nitrate reductase enzyme. Then, the produced nitrite was assayed using Griess reagent at an optical density of 550 nm.

#### Estimation of SIRT1, IL-17, NF-κB, IKBKB, PPARγ, COX-2, TNF-α, and PKC

Lung contents of SIRT1, IL-17, NF-κB, IKBKB, PPARγ, COX-2, TNF-α, MPO, and PKC were determined using ELISA kits. Standards and samples were pipetted into wells with immobilized antibodies specific for rat SIRT1, IL-17, NF-κB, IKBKB, PPARγ, COX-2, TNF-α, MPO, and PKC and then were incubated for 30 min at 37 °C. After incubation and washing, horseradish peroxidase-conjugated streptavidin was pipetted into the wells, and incubated for 30 min at 37 °C, which was washed once again. Tetramethylbenzidine (TMB) substrate solution was added to the wells and incubated for 15 min at 37 °C; a color was developed proportionally to the amount of SIRT1, IL-17, NF-κB, IKBKB, PPARγ, COX-2, TNF-α, MPO, and PKC bound. Color development was discontinued (stop solution) and after 10-min color intensity was measured at 450 nm (Salama et al. 2021).

#### Quantitative real-time PCR gene expression of Nrf2 and FoxO1 in rats` lung tissues

Rats` lung tissue homogenates were used for total RNA extraction by RNeasy Purification Reagent (Promega, Madison, WI, USA) according to the manufacturers’ protocol. The extracted RNA was then quantified by spectrophotometry (JENWAY, USA) at 260 nm. The obtained RNA is then used to assess the expression levels of *Nrf2 and FoxO1 *genes with quantitative RT-PCR based on the SYBR Green (TransScript^™^ II Green One-Step qRT-PCR SuperMix; TransGen. Cycling conditions were adjusted as described by the manufacturer. PCR primers were designed with Gene Runner Software (Hastings Software Inc., Hastings, NY, USA) with RNA sequences obtained from GenBank, using Beta Actin as a housekeeping gene (Table1). The obtained data were analyzed using the 2^−ΔΔ^ CT method (Livak and Schmittgen 2001).

**Table 1:** Primer sequences used for RT-qPCR

Gene	Forward primer	Reverse primer
Nrf2	5′-TTGTAGATGACCATGAGTCGC-3′	5′-TGTCCTGCTGTATGCTGCTT-3′
FoxO1	5′-GGGTCCCACAGCAACGATG-3′	5′-CACCAGGGAATGCACGTCC-3′
Beta actin	5′-AGGTCGGAGTCAACGGATTTGGT-3′	5′-CATGTGGGCCATGAGGTCCACCAC-3′

### Histological examination

Various groups' lung tissue was fixed in 10% formalin after being dissected. After being fixed for a day or two, the tissue was dehydrated progressively by stronger alcohol (70%, 90%, and three changes in absolute alcohol), cleared with xylene, impregnated in three changes of soft paraffin wax at 50 °C, and lastly embedded in paraffin wax to form solid blocks. A 7-μm thick transverse section was cut in a serial cross-section. Hematoxylin and eosin were used to stain paraffin slices mounted on glass slides with a layer of albumin glycerin. Light microscopy was used to qualitatively analyze the stained tissue slices (Carleton et al. 1980).

### Histomorphometric analysis

Based on the previous studies, quantify the extent of inflammatory infiltration and tissue damage (Patel et al. 2012). Whole-lung sections were scored by observers who were blinded for groups. The degree of inflammatory cell infiltration and bronchiolar epithelium hyperplasia were assessed in alveolar septa and lumens. The scores were derived using light microscopy and scored based on the intensity of alterations: 0, absent; 1, mild; 2, moderate; 3 severe. Scores of rats from the same group were pooled and calculated as mean ± standard deviation of the mean (SD).

### Statistical analysis

All the values are presented as means ± SD. The data of this study were evaluated by one-way analysis of variance followed by Tukey’s multiple comparisons test. Graph pad Prism software, version 5 (Inc., San Diego, USA) was used to carry out these statistical tests. The difference was considered significant when *P* < 0.05.

## Results

### Effect of protocatechuic acid on PPARγ, COX-2, NF-κB, and IKBKB protein levels

**Fig. 1:**
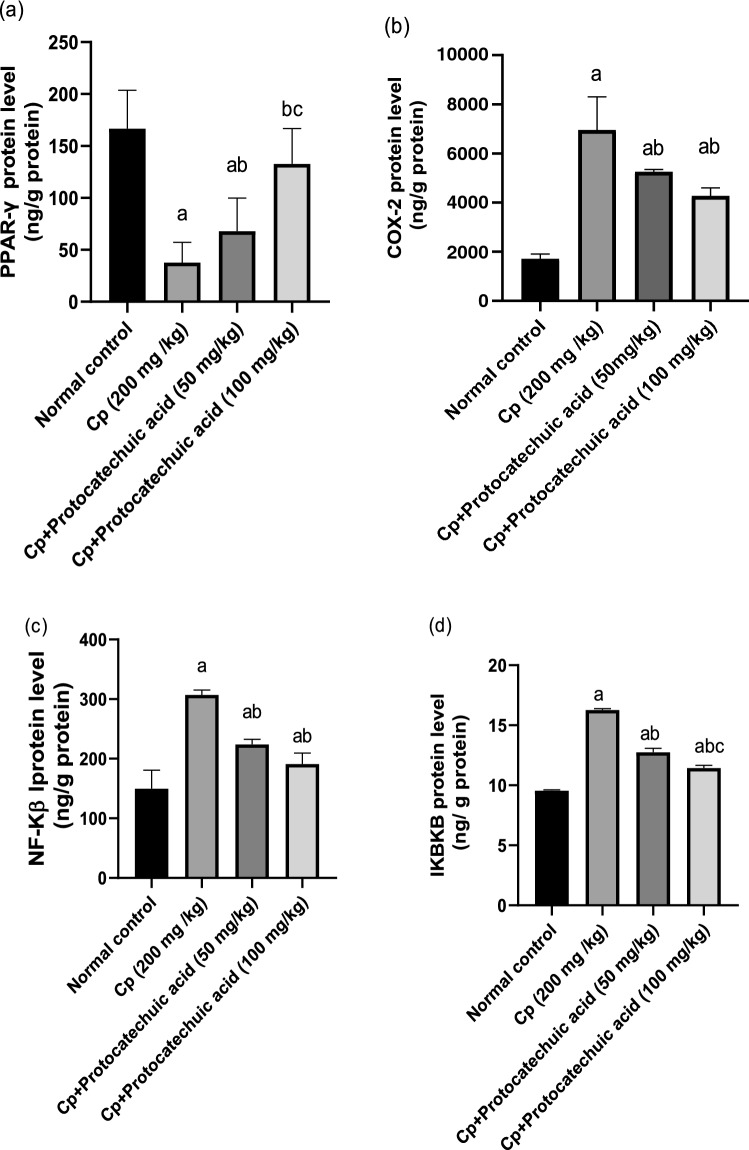
Effect of protocatechuic acid on PPARγ, COX-2, NF-Κβ, and IKBKB proteins levels. The effect of treatment with protocatechuic acid (50, 100 mg/kg; p.o.) once daily for 10 consecutive days after cyclophosphamide (200 mg/kg; i.p.) single dose on **a** PPARγ **b** COX-2 **c** NF-Κβ **d** IKBKB proteins levels. The data were expressed as mean ± SD, (*n* = 8). Statistical analysis was carried out by one-way ANOVA followed by Tukey HSD test for multiple comparisons. ^a^Significantly different from normal control. ^b^Significantly different from cyclophosphamide control. ^c^Significantly different from cyclophosphamide + protocatechuic acid 50 mg/kg at P < 0.05

CP injection significantly decreased the lung protein levels of PPARγ by 77% and increased COX-2 by 305% respectively, as compared to control group (*P* value < 0.05). Meanwhile, in protocatechuic acid 50 and 100 mg/kg significantly increased PPARγ by 79 and 250% as well as reduced COX-2 levels by 24 and 39% respectively, as compared to the CP-treated group (*P* value < 0.05). Moreover, CP treatment significantly increased NF-κB and IKBKB lung protein levels by 105 and 70.3% respectively, in comparison with the control group. However, administration of protocatechuic acid 50 and 100 mg/kg significantly decreased NF-κB by 27 and 38%, along with IKBKB by 22 and 30% respectively, when compared with that of the CP group (*P* value < 0.05) (Fig. 1).

### Effect of protocatechuic acid on IL-17 protein levels

**Fig. 2:**
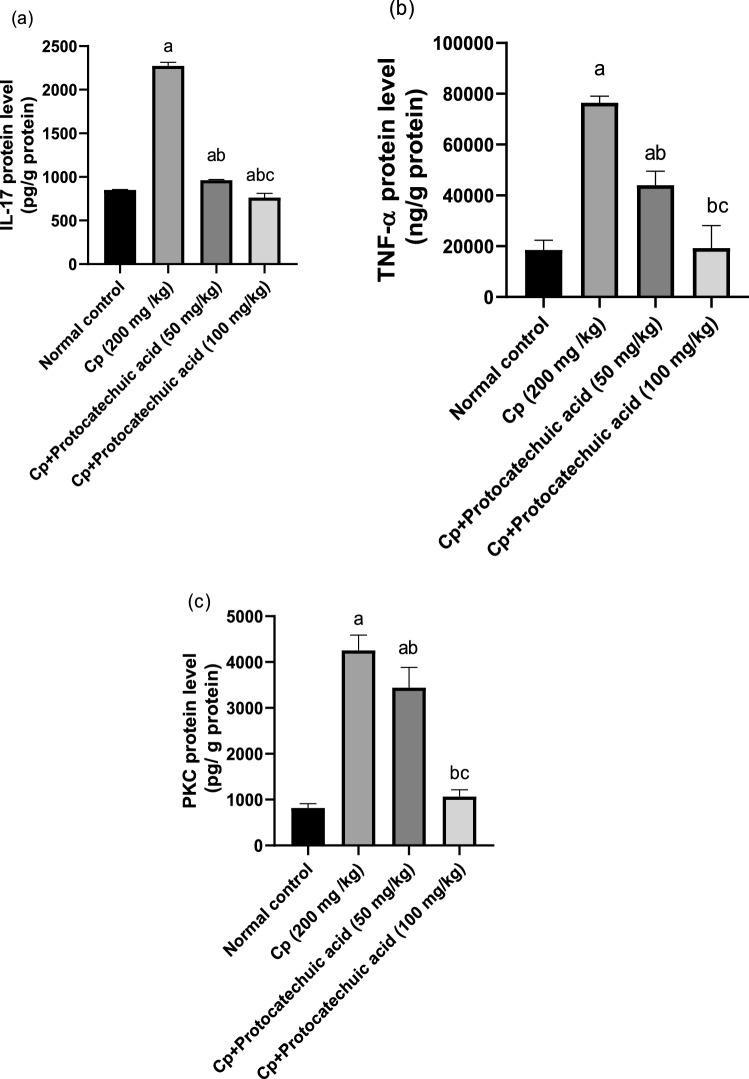
Effect of protocatechuic acid on IL-17, TNF-α, and PKC proteins levels. The effect of treatment with protocatechuic acid (50, 100 mg/kg; p.o.) once daily for 10 consecutive days after cyclophosphamide (200 mg/kg; i.p.) single dose on **a** IL-17 **b** TNF-α **c** PKC proteins levels. The data were expressed as mean ± SD, (*n* = 8). Statistical analysis was carried out by one-way ANOVA followed by Tukey HSD test for multiple comparisons. ^a^Significantly different from normal control. ^b^Significantly different from cyclophosphamide control. ^c^Significantly different from cyclophosphamide + protocatechuic acid 50 mg/kg at *P* < 0.05

CP treatment significantly increased IL-17 lung protein levels by 167% in comparison with the control group (*P* value < 0.05). However, administration of protocatechuic acid 50 and 100 mg/kg significantly decreased IL-17 by 58 and 66%, respectively, when compared with that of the CP group (*P* value < 0.05). The treatment with 100 mg/kg of protocatechuic acid reduced the contents of IL-17 to their normal value, compared to the CP group (Fig. 2a).

### Effect of protocatechuic acid on TNF-α and PKC protein levels

Lung protein levels of TNF-α and PKC were significantly raised by 312 and 410% respectively, compared to the control rats (*P* value < 0.05). While, in protocatechuic acid 50 and 100 mg/kg caused a significant decline in the TNF-α level by 42 and 75% besides the diminution of PKC levels by 19 and 75%, respectively, as compared to CP-treated group (*P* value < 0.05). Treatment of protocatechuic acid 100 mg/kg reduced pulmonary levels of TNF-α to their normal value, compared to the CP group (Fig. 2b, c).

### Effect of protocatechuic acid on SIRT1 protein level

**Fig. 3:**
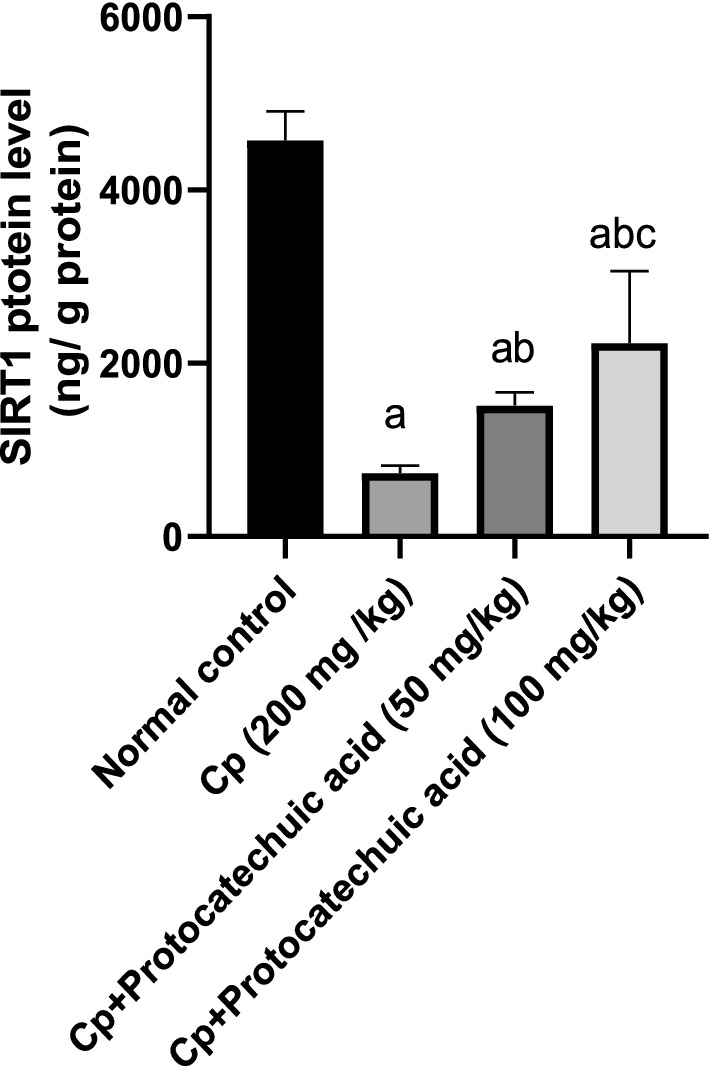
Effect of protocatechuic acid on SIRT1 proteins level. The effect of treatment with protocatechuic acid (50, 100 mg/kg; p.o.) once daily for 10 consecutive days after cyclophosphamide (200 mg/kg; i.p.) single dose on SIRT1 proteins level. The data were expressed as mean ± SD, (*n* = 8). Statistical analysis was carried out by one-way ANOVA followed by Tukey’s HSD test for multiple comparisons. ^a^Significantly different from normal control. ^b^Significantly different from cyclophosphamide control. ^c^Significantly different from cyclophosphamide + protocatechuic acid 50 mg/kg at *P* < 0.05

CP injection significantly decreased the lung protein level of SIRT1 by 84% compared to the control group (*P* value < 0.05). Meanwhile, in protocatechuic acid 50 and 100 mg/kg SIRT1 protein levels were significantly increased the by 87 and 205% respectively, as compared to the CP-treated group (*P* value < 0.05) (Fig. 3).

### Effect of protocatechuic acid on GSH, catalase, MDA, NO, and MPO pulmonary protein levels

**Fig. 4:**
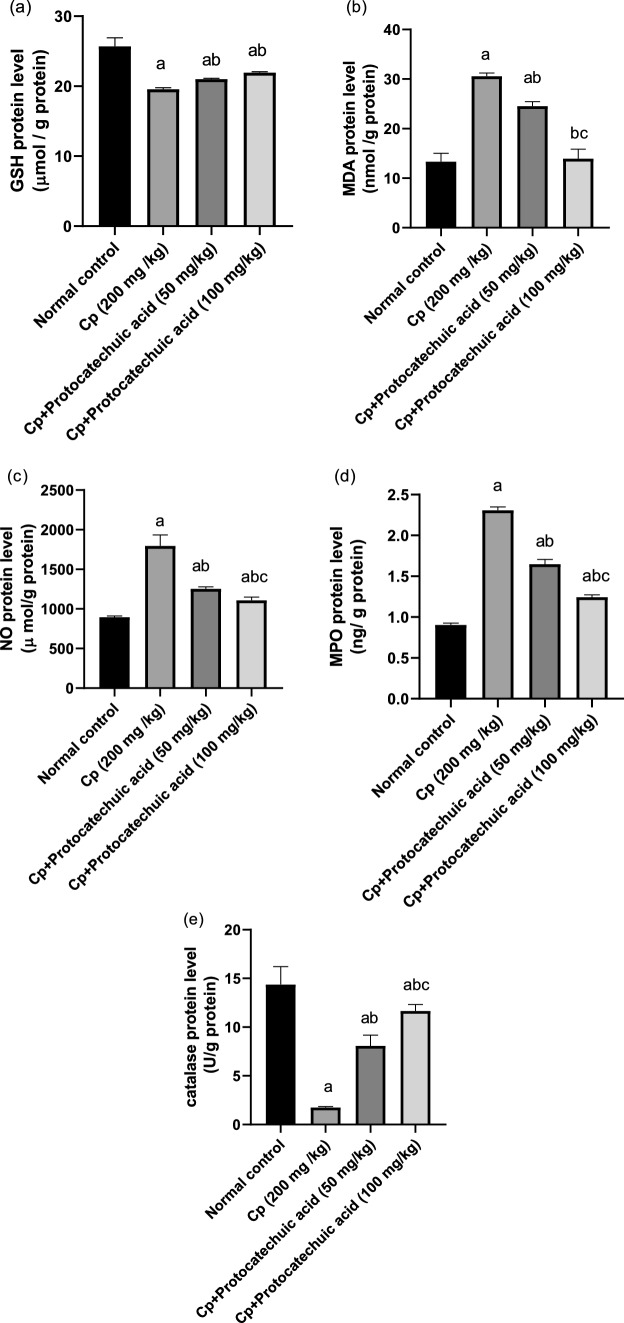
Effect of protocatechuic acid on GSH, MDA, NO, MPO, and catalase proteins levels. The effect of treatment with protocatechuic acid (50, 100 mg/kg; p.o.) once daily for 10 consecutive days after cyclophosphamide (200 mg/kg; i.p.) single dose on **a** GSH **b** MDA **c** NO **d** MPO AND **e** catalase proteins levels. The data were expressed as mean ± SD, (*n* = 8). Statistical analysis was carried out by one-way ANOVA followed by Tukey HSD test for multiple comparisons. ^a^Significantly different from normal control. ^b^Significantly different from cyclophosphamide control. ^c^Significantly different from cyclophosphamide + protocatechuic acid 50 mg/kg at *P* < 0.05

The protein levels of GSH and catalase were significantly lower in the CP group by 24 and 88% respectively, as compared to control group (*P* value < 0.05). The levels of MDA, NO and MPO were significantly higher in CP group by 98, 100 and 156%, respectively, than the control group (*P* < 0.05). Although protein levels of GSH and catalase were significantly higher in protocatechuic acid 50 and 100 mg/kg by 8, 12.3 and 371, 457% respectively, when compared with CP-treated group (*P* value < 0.05). Moreover, protocatechuic acid 50 and 100 mg/ kg significantly decreased the levels of MDA, NO and MPO by 20, 54 and 30%, 38, 29, 48%, respectively, when compared with CP-treated group (*P* value < 0.05) (Fig. 4).

### Effect of protocatechuic acid on FoxO-1 and Nrf-2 gene expression

**Fig. 5:**
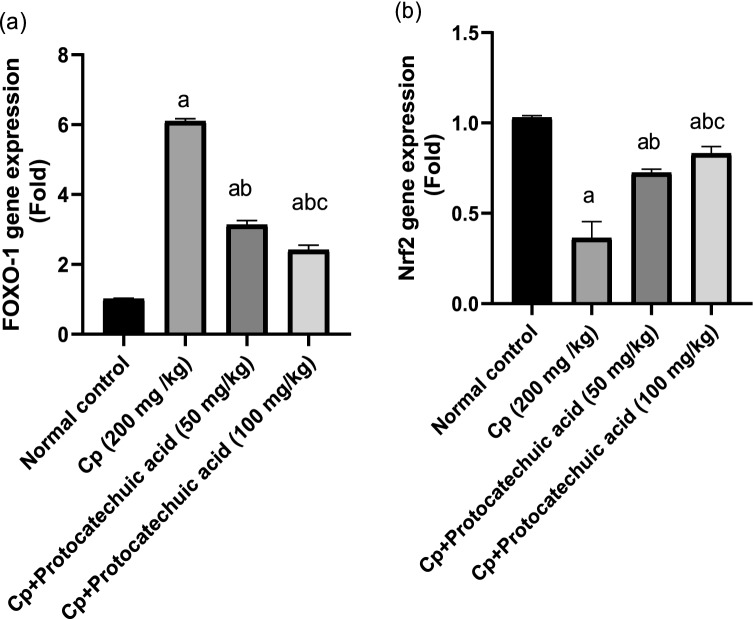
Effect of protocatechuic acid on FoxO-1 and Nrf-2 gene expression. The effect of treatment with protocatechuic acid (50, 100 mg/kg; p.o.) once daily for 10 consecutive days after cyclophosphamide (200 mg/kg; i.p.) single dose on **a** FoxO-1 **b** Nrf-2 gene expression. The data were expressed as mean ± SD, (*n* = 8). Statistical analysis was carried out by one-way ANOVA followed by Tukey HSD test for multiple comparisons. ^a^Significantly different from normal control. ^b^Significantly different from cyclophosphamide control. ^c^Significantly different from cyclophosphamide + protocatechuic acid 50 mg/kg at *P* < 0.05

Administration of CP showed a significant decrease in the Nrf-2 gene (*P* value < 0.05) with a marked increase in foxo-1, when compared with the control group (*P* value < 0.05). Although protocatechuic acid 50 and 100 mg/kg administration resulted in a significant increase in Nrf-2 compared with cyclophosphamide group (*P* value < 0.05), moreover, the Foxo-1 gene was downregulated appreciably in a dose-dependent manner as compared to the CP group (*P* value < 0.05) (Fig. 5).

### Histopathological study

**Fig. 6:**
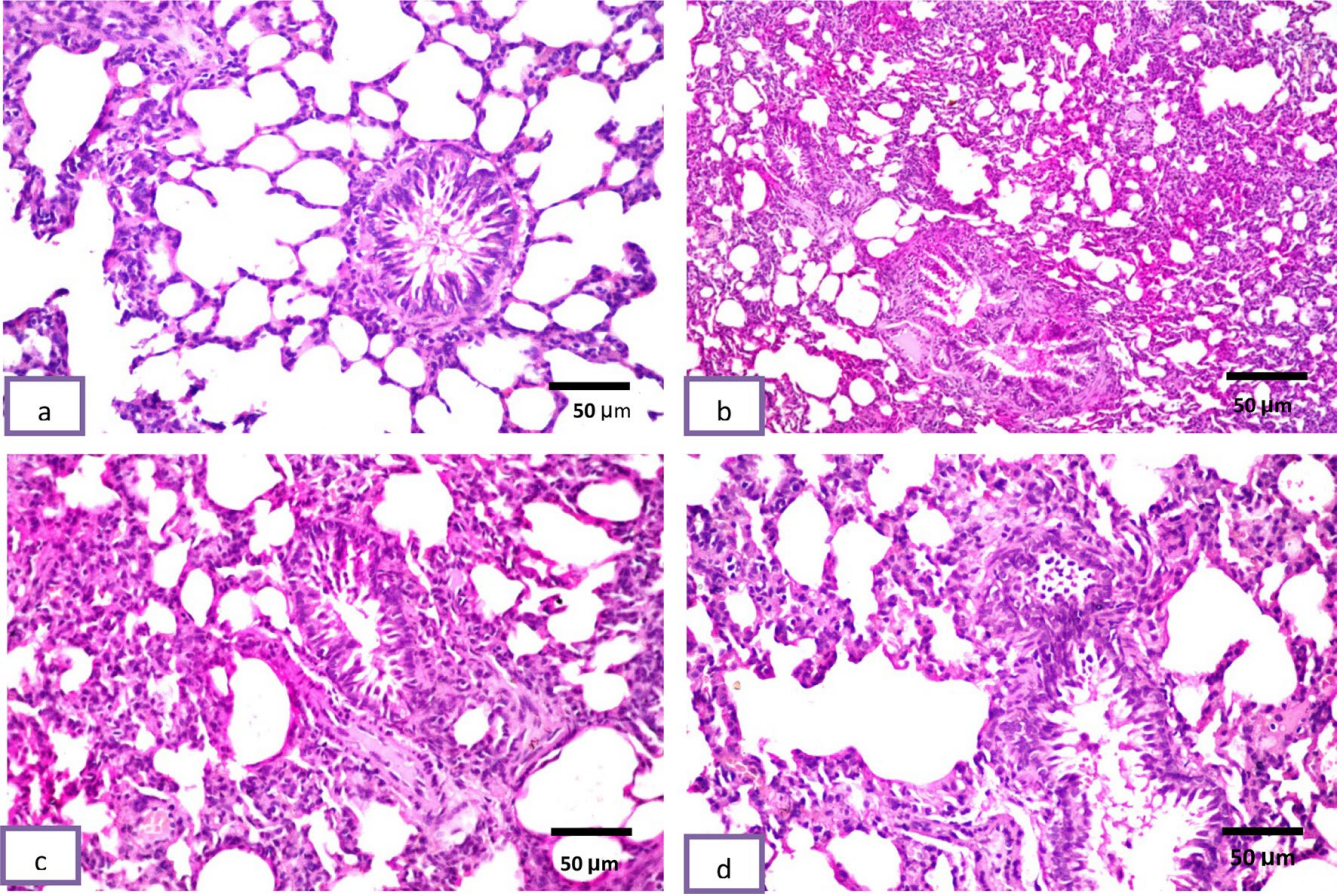
Effect of treatment with protocatechuic acid on lung histopathology. **a** Lung sections from normal control rats showed no histopathological alteration and the normal histological structure of the bronchioles and the surrounding air alveoli were recorded. **b** Cyclophosphamide group showed collapse and emphysema were detected in the air alveoli associated with bronchiolar epithelium hyperplasia and inflammatory cells infiltration in between all. **c** Protocatechuic acid 50 group showing congestion in the blood vessels associated with emphysema of the air alveoli. The bronchioles showed moderate epithelial lining hyperplasia with inflammatory cells infiltration in the bronchiolar lumen. **d** Protocatechuic acid 100 group showed less inflammatory cells infiltration in the bronchiolar lumen (H&E X 200)

Histological pictures of lung sections from normal control rats showed no histopathological alteration and the normal histological structure of the bronchioles and the surrounding air alveoli were recorded in (Fig. 6a). The CP group showed collapse and emphysema detected in the air alveoli associated with bronchiolar epithelium hyperplasia and inflammatory cells infiltration in between all (Fig. 6b). Protocatechuic acid 50 group show congestion in the blood vessels associated with emphysema of the air alveoli. The bronchioles showed moderate epithelial lining hyperplasia with inflammatory cells infiltration in the bronchiolar lumen (Fig. 6c). In the protocatechuic acid 100 group the bronchioles showed less inflammatory cells infiltration in the bronchiolar lumen (Fig. 6d).

### Histomorphometric results

**Fig. 7:**
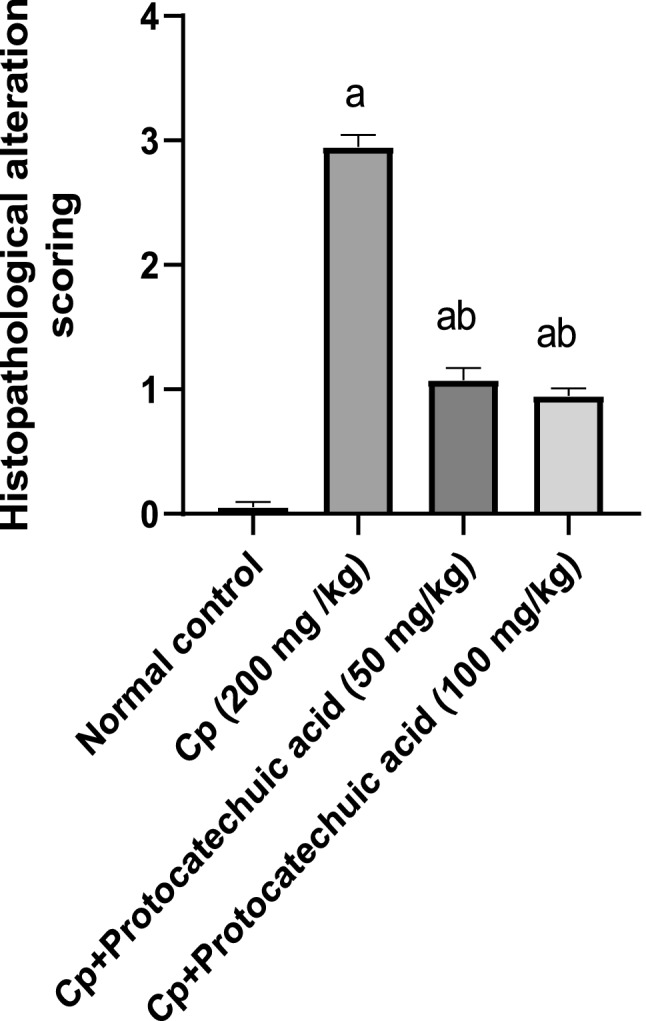
Effect of treatment with protocatechuic acid on histomorphometric analysis. The data were presented as the mean ± SD of (*n* = 4) for each group. Statistical analysis was carried out by one-way analysis of variance followed by Tukey’s multiple comparisons test. ^a^Statistically significant from the control group. ^b^Statistically significant from the cyclophosphamide group at *P* < 0.05

As shown in Fig. 7, results of negative control showed normal brain architecture. Conversely, CP group exhibited severe histopathological alterations, such as degree of inflammatory cell infiltration and bronchiolar epithelium hyperplasia. Interestingly, protocatechuic acid 50 and 100 groups significantly showed moderate and mild inflammatory cell infiltration and bronchiolar epithelium hyperplasia, respectively, as compared to the CP group.

## Discussion

Peroxisome proliferator-activated receptors (PPARs) are nuclear hormone and regulate the genes expression implicated in several biological processes (Corton et al. 2000; Vamecq and Latruffe 1999). PPAR mediated transcription factors NF-κβ inhibition and enhanced IκBα expression (Gervois et al. 2000) leading to reduced inflammatory cell influx and injury to the alveolar capillary and attenuated chemokine, cytokine, adhesion molecule expression and eicosanoid production(Ishii et al. 2000). Moreover, it has suppressive effect on many proinflammatory gene expression as IL-6 and COX-2 (Delerive et al. 1999).

In the current study, CP decreased PPARγ with an elevation in NF-κB/COX-2 lung contents when compared with normal group, while PCA treatment can be attributed to stimulated PPARγ and reduced NF-κB/COX-2 lung contents as compared to CP-treated group. These present experimental findings demonstrate the promising anti-inflammatory and lung protective activity of PCA. Previously, PCA had anti-inflammatory and analgesic activity against carrageenan via inhibition of arthritis index and the synthesis or release of prostaglandins (Lende et al. 2011). Moreover, protective effects of PCA have been reported on doxorubicin-induced kidney damage as it is considered as iNOS and COX2 inhibitor (Molehin et al. 2019).

In addition, T-helper (Th)-17 cells induced the production of pro-inflammatory IL-17 (Ghoreschi et al. 2010), and are implicated in the pathogenesis of lung disease (Yanagisawa et al. 2017), and their activation in the presence of a combination of TGF-β, IL-6, and IL-1β mediated these pro-inflammatory differentiation (Ghoreschi et al. 2010). IL-6 with TGF-β induces Th17 development by activating iNOS and stimulating the expression of macrophage, IL-1 β, IL-6, TNF-α, and several chemokines, which potentiates the inflammatory response (Gonçalves-de-Albuquerque et al. 2017). The present study showed that PCA decreased IL-17 with the inhibition of NF-κB/IKBKB and TNF-α/PKC lung contents when compared with lung injury group. PCA, in another study, attenuated cerebral aneurysm progression by blocking the activation of TNF-α/NF-κB/ IL-17 signaling pathways (Molehin et al. 2019). Moreover, PCA suppressed inflammation and apoptosis in lung injury induced by LPS via modulating TNF-α, NF-κB, and IL-1β in lung tissue (Alsharif et al. 2021).

Another vital molecule in pulmonary damage is SIRT1, which has antioxidative and anti-inflammatory effects (Bai et al. 2015). SIRT enhances mitochondrial function, inhibiting the release of ROS (Yang et al. 2021). A high level of SIRT1 was associated with GSH and CAT overexpression (Ding et al. 2016). Moreover, SIRT1 protected H_2_O_2_-induced senescent endothelium (Ren et al. 2019). The present results revealed that CP suppressed SIRT1 lung content with a decrease in GSH and CAT as well as an elevation in NO, MDA (lipid peroxidation), and MPO (indicator of neutrophil accumulation), while treatment with PCA activated SIRT1 enhancing cellular responses to oxidative stress via upregulation of GSH, CAT as well as downregulation of NO, MDA, MPO, reducing acute pulmonary damage in this rat model. Previously, PCA regulated the SIRT1/NF-κB pathway in brain microglial activation induced by lipopolysaccharide (LPS) (Kaewmool et al. 2020). Moreover, FoxO1 is one of the family members of forkhead box protein (FoxO), it is a transcription factor that regulates several biological processes such as oxidative stress, cell cycle arrest, and apoptosis (Kim et al. 2017).

FoxO1 is one of the substrates of SIRT1 which deacetylates FoxO1 (Lee et al. 2018), leading to its inactivation (Sedding and Haendeler 2007) mediating its pro-survival action, while the acetylation of FoxO1 enhances the apoptotic action (Papanicolaou et al. 2008), and oxidative injury (Elrashidy and Hasan 2021). The recent studies exploring the oxidative stress due to CP administration showed it decreased the expression of SIRT1(Salama et al. 2020), which in turn decreases both phosphorylated and deacetylated FoxO1 and in turn enhanced FoxO1 gene expression (Dong et al. 2021; Elrashidy and Hasan 2021). In agreement with the previous research, our results revealed that administration of CP markedly increased FoxO-1 expression, whereas, PCA decreased it. Previously, PCA can phosphorylate several substrates, including FoxO1 (Tia et al. 2018), mitigating oxidative stress, and inhibiting apoptosis (Kaewmool et al. 2020; Salama et al. 2020). Thus, simultaneously targeting FoxO1 and SIRT1 will greatly impact the alleviation of pulmonary damage associated with organ toxicity and promotion of cell survival.

Pulmonary damage induced by CP also associated with oxidative stress and a reduction of nuclear factor E2-related factor 2 (Nrf2) transcription factor that regulate the cellular antioxidative responses by promoting the expression of series of antioxidant genes (Tonelli et al. 2018). Our results exhibited a significant decrease in Nrf2 gene expression in the CP- treated group, in accordance with previous studies showing decreased levels of Nrf2 in response to CP administration (Aladaileh et al. 2019; ALHaithloul et al. 2019). On the other hand, our results demonstrated a significant increase in Nrf2 gene expression in PCA-treated groups compared to the CP group in a dose dependent manner, this was supported by results of previous research (Ibitoye and Ajiboye 2020). Revealing that PCA enhanced Nrf2 expression, also, Je and Lee (2015) reported that a plant extract containing PCA activated Nrf2. Together, these findings imply that PCA can reduce the oxidative stress and pulmonary damage brought on by CP, as indicated by the increased expression of Nrf2.

## Conclusion

The current study exhibited that PCA has a vital role in cellular inflammatory, oxidative, and immune responses through modulating PPARs/IL-17/Nrf2 to mitigate CP-induced acute lung injury in the rat. In addition, PCA regulates the SIRT1/FoxO1 pathway that helps in the treatment of lung inflammatory diseases. Further investigations are required to prove PCA's ability to prevent pulmonary damage associated with CP in the clinical setting and using it in a treatment regime will be highly worthwhile.

## Data Availability

The data presented in this study are available within the text and figures of the paper. Original data are available from the corresponding authors.
